# Overlap of burnout-depression symptoms among Chinese neurology graduate students in a national cross-sectional study

**DOI:** 10.1186/s12909-021-02511-3

**Published:** 2021-02-02

**Authors:** Wei Zhou, Juncai Pu, Xiaogang Zhong, Wensong Yang, Teng Teng, Li Fan, Haiyang Wang, Lu Tian, Yiyun Liu, Peng Xie

**Affiliations:** 1 School of Public Health and Management, Chongqing Medical University, Chongqing, 400016 China; 2 NHC Key Laboratory of Diagnosis and Treatment on Brain Functional Diseases, The First Affiliated Hospital of Chongqing Medical University, Yuzhong District, Chongqing, China; 3 Department of Neurology, The First Affiliated Hospital of Chongqing Medical University, Chongqing, 400016 China; 4 Key Laboratory of Psychoseomadsy, Stomatological Hospital of Chongqing Medical University, Chongqing, 401147 China; 5 Stomatological Hospital of Chongqing Medical University, Chongqing, 401147 China; 6 China Neurologist Association of Chinese Medical Doctor Association, Beijing, 100010 China

**Keywords:** Overlap, Neurology, Postgraduates, Burnout, Depressive symptoms

## Abstract

**Background:**

The overlap of burnout and depression is a phenomenon that can effectively reflect the psychological state of a group. However, whether burnout is a type of depression is still debated in current research. The high incidence of burnout and depressive symptoms among medical students indicates that it is urgent to provide appropriate health services for them. However, the proportion of burnout and depression in the overlapping symptoms experienced by medical students, and the characteristics of the relative influencing factors, remain unclear. Therefore, we addressed these issues for neurology graduate students in China.

**Methods:**

Using data from a cross-sectional survey of Chinese neurology graduate students, a diagnostic model was established according to their burnout and/or depression symptoms. Burnout was assessed by using the Maslach Burnout Inventory. Depression symptoms were assessed with a two-item depression screening tool for primary care evaluation of mental disorders. Univariate analyses with chi-squared tests were conducted to assess associations between variables. Multinomial logistic regression models were used to analyze the effects of multiple factors on dependent variables. The factors included demographic information and three medical-study related problems.

**Results:**

In total, 32.2% of surveyed students evidenced overlapping burnout and depression symptoms. Students with depressive symptoms tended to be included in the burnout students’ category. In the regression model, being unmarried, having children, and career choice regret were related to students who had only burnout, while the students with overlapping symptoms were affected by more factors such as family income, the consideration of dropping out once.

**Conclusions:**

The symptoms and related factors of burnout and depression among Chinese neurology postgraduates have obvious overlap and show a significant trend. The occurrence of depressive symptoms among medical students is closely related to whether they are burned out. Students with only burnout were common, but students with only depressive symptoms were uncommon. Finally, burnout may be a pre-depression state.

**Supplementary Information:**

The online version contains supplementary material available at 10.1186/s12909-021-02511-3.

## Background

Burnout includes three dimensions of exhaustion, cynicism and lack professional efficacy [1, 2]. It is a more contextualized mental health problem and is thought to be related to depressive symptoms in the work environment [3, 4]. Depression symptoms include low mood, anhedonia, fatigue overlap with burnout and there is a strong correlation between them [5]. Burnout and depressive symptoms were determined to be influenced by common factors, such as work stress [6, 7]. The study of their overlap is a highly contentious area.

Reports around the world have increasingly pointed out that medical students’ experience high levels of burnout and depressive symptoms [8–11]. A study that covered 43 countries in 2016 showed prevalence of depressive symptoms among medical students was 27.2% [12], and another study reported in 2019 showed that the incidence of burnout among medical students was 44% [13]. Burnout or depressive symptoms of medical students were affected by career choice motivation, poor economic status, relatives and other dangerous factors [14, 15]. At the same time, it has been shown to impact students extremely negatively, producing self-reported unprofessional conduct, less altruistic professional values, declining interest in a medical career, increasing suicidal ideation, among other outcomes [16–18]. Overall, the cited studies highlight that burnout and depressive symptoms are important issues affecting the mental health of medical students. Our previous studies have discussed the characteristics of burnout and depressive symptoms among Chinese neurology graduate students, respectively [19, 20]. While these findings have improved our understanding of medical students’ mental health issues, more recent studies have begun to focus on the overlap between burnout and depressive symptoms, and have offered judgments that better reflect the social reality [7, 21]. To this end, some scholars have investigated this overlap relationship by using input from physicians, school administrators, and health professionals [22–24]. They have shown that, in their study populations, burnout and depressive symptoms were indivisible and, furthermore, were probably the same symptoms [22–24].

Another study showed that individuals with severe burnout were significantly more likely to use antidepressants [25]. This suggests that burnout is worthy of attention in clinical practice. Thus, re-examining burnout and depressive symptoms can provide a basis for further identification and treatment. Importantly, the nature of medical education has made medical students to pay more attention to the stigma of mental illness. And the effects of burnout, depression, and suicidal ideation on medical students is more serious than that on the general population [26].

To our knowledge, there are few studies on the overlap between burnout and depressive symptoms among medical students. Moreover, the degree and the risk factors of overlap are not clear. The aim of this study was to investigated the features of overlapping symptoms of depression and burnout in a large sample of Chinese neurology graduate students, and further established a corresponding diagnostic model.

## Methods

### Participants

Using a cross-sectional study design, a national self-administered questionnaire survey was conducted among 1814 neurology postgraduates in China from September 2014 to March 2015 [19, 20, 27]. Participants were recruited from 249 hospitals in 27 provinces across the country [19, 20]. Survey responses were voluntary and anonymous. Consent was assumed for any participant who returned a completed survey. All methods were performed in accordance with the relevant guidelines and regulations. The study was reviewed and granted approval by the Ethics Committee of Chongqing Medical University.

### Outcome measures

Validated surveys were used to measure burnout and depressive symptoms. Demographic data and three questions related to studying medicine were collected to measure factors associated with burnout and depressive symptoms. Demographic characteristics comprised gender, academic year, family monthly income, type of degree, postgraduate entrance examination score, hours worked or studied per week, hours slept per day, marital status, whether have children, and part-time work experience [19]. The three questions related to studying medicine were: “If you could go back, would you choose to become a doctor again?”; “What do you think of the current medical environment?”; and, “Have you ever considered dropping out of school?”

The Maslach Burnout Inventory was used to evaluate burnout; its three dimensions are: emotional exhaustion (9 items), depersonalization (5 items), and personal accomplishment (8 items) [1, 28]. Participants rated their answers on a 7-point frequency scale (0 = Never, 1 = a few times a year or less, 2 = once a month or less, 3 = several times a month, 4 = once a week, 5 = a few times a week, 6 = every day). Respondents were considered to have burnout if they scored higher on the emotional exhaustion (≥27) and/or depersonalization (≥10) subscales [16].

The two-item primary care evaluation of mental disorders (PRIME-MD) was used to assess depressive symptoms [29]. Participants’ positive response to either of the following items was considered evidence of depressive symptoms: “In the last month, did you often feel in low spirits, depressed or hopeless?” and, “In the last month, were you often troubled by the feeling that you were not interested or had no fun in doing things?” PRIME-MD has been widely adopted for the assessment of depression symptoms in medical students [30, 31].

### Statistical analysis

Data analyses were conducted by using IBM SPSS version 21(IBM Corp., Armonk, N.Y., USA). Data were divided into four categories according to participants’ scores: 1) no burnout or depressive symptoms, 2) burnout but no depressive symptoms, 3) depressive symptoms but no burnout, 4) both burnout and depressive symptoms. Univariate analyses with chi-squared tests were conducted to assess associations between variables. All variables with *p* ≤ 0.1 in the univariate analysis were included in the multinomial logistic regression model, and the significance level (alpha) of variable selection was set at 0.05 for both entry and exit in the logistic regression models. Multicollinearity analysis was used to test collinearity among variables when the tolerance was ≤0.1 or the variance inflation factor was ≥10 [19]. The significance level was set at 0.05.

## Results

### Descriptive analysis

The demographics, the answers to three questions about studying medicine, and mental health characteristics of the sample are summarized in Table 1. Overall, 51.4% of students evidenced burnout alone, 1.5% suffered from depressive symptoms alone, and 32.2% had both burnout and depression symptoms. Both clinical degree and women made up more than 50% of the students in four categories. In addition, less than 10% of students in each category felt the medical environment was good. In the univariate analysis of multiple groups, academic year, family monthly income, postgraduate entrance examination score, hours slept per day, marital status, whether have children, view on current medical environment, career choice regret, and considered dropping out once were statistically different between groups (*p* <  0.05).

**Table 1:** Characteristics associated with medical students’ burnout and (or) depressive symptoms

Characteristics	Burnout and Depressive Symptoms				***P*** Value
	B (−) and D (−) [271, 14.9%]	B (+) and D (−) [932, 51.4%]	B (−) and D (+) [27, 1.5%]	B (+) and D (+)[584, 32.2%]	
**Gender, Female**	172_a_(63.5)	640_a_(68.8)	14_a_(51.9)	392_a_(67.2)	0.13
**Academic year**					< 0.01
First-year, master’s degree	73_a_(27.7)	216_a_(23.6)	1_a,b_(4.3)	101_b_(17.7)	
Second-year, master’s degree	69_a_(26.1)	269_a_(29.4)	8_a,b_(34.8)	208_b_(36.4)	
Third-year, master’s degree	101_a_(38.3)	311_a_(34.0)	8_a_(34.8)	189_a_(33.1)	
First-year, doctor’s degree	7_a_(2.7)	43_a_(4.7)	3_a_(13.0)	26_a_(4.6)	
Second-year, doctor’s degree	8_a_(3.0)	37_a_(4.0)	3_a_(13.0)	26_a_(4.6)	
Third-year, doctor’s degree	6_a_(2.3)	40_a_(4.4)	0_a_(0.0)	22_a_(3.9)	
**Degree type**					0.09
Clinical practice	194_a_(72.7)	619_a_(67.9)	15_a_(57.7)	415_a_(72.7)	
Academic practice	73_a_(27.3)	292_a_(32.1)	11_a_(42.3)	156_a_(27.3)	
**Family income (yuan per month)**					0.02
< 5000	145_a,b_(53.7)	434_b_(46.8)	9_a,b_(33.3)	313_a_(54.1)	
5000–10,000	84_a_(31.1)	315_a_(33.9)	11_a_(40.7)	193_a_(33.3)	
10,000–15,000	22_a_(8.1)	103_a_(11.1)	5_a_(18.5)	46_a_(7.9)	
> 15,000	19_a,b_(7.0)	76_b_(8.2)	2_a,b_(7.4)	27_a_(4.7)	
**Scores of postgraduate entrance examination**					< 0.01
< 300	54_a_(23.2)	93_b_(11.5)	1_a,b_(4.3)	73_a,b_(15.6)	
300–330	84_a_(36.1)	380_b_(47.0)	9_a,b_(39.1)	225_b_(48.0)	
330–360	65_a_(27.9)	229_a_(28.3)	10_a_(43.5)	118_a_(25.2)	
> 360	30_a_(12.9)	106_a_(13.1)	3_a_(13.0)	53_a_(11.3)	
**Hours worked or studied per week (h)**					0.05
< 35	23_a_(8.6)	65_a_(7.0)	5_a_(18.5)	39_a_(6.7)	
35–45	68_a_(25.4)	221_a_(23.8)	7_a_(25.9)	132_a_(22.8)	
45–55	88_a_(32.8)	266_a_(28.6)	8_a_(29.6)	150_a_(25.9)	
> 55	89_a_(33.2)	377_a,b_(40.6)	7_a,b_(25.9)	259_b_(44.7)	
**Hours slept per day (h)**					< 0.01
< 6	30_a,b_(11.1)	96_b_(10.3)	3_a,b_(11.1)	95_a_(16.3)	
6–8	219_a_(80.8)	767_a_(82.5)	19_a_(70.4)	457_a_(78.3)	
8–10	22_a,b_(8.1)	67_a,b_(7.2)	5_b_(18.5)	32_a_(5.5)	
**Marital status, Married**	58_a_(21.4)	136_b_(14.7)	7_a,b_(25.9)	101_a,b_(17.3)	0.03
**Have children**	65_a_(24.0)	289_a,b_(31.0)	11_a,b_(40.7)	223_b_(38.3)	< 0.01
**Have worked part-time once**	92_a_(34.5)	342_a_(36.8)	15_a_(55.6)	238_a_(41.0)	0.05
**View on current medical environment**					< 0.01
Poor	116_a_(43.0)	493_b_(53.1)	8_a,b_(29.6)	426_c_(73.3)	
Neutral	145_a_(53.7)	414_a_(44.6)	17_a_(63.0)	135_b_(23.2)	
Good	9_a_(3.3)	21_a_(2.3)	2_a_(7.4)	20_a_(3.4)	
**Career choice regret**					< 0.01
With	75_a_(27.7)	408_b_(43.8)	9_a,b_(33.3)	353_c_(60.7)	
Neutral	43_a_(15.9)	191_a_(20.5)	4_a_(14.8)	91_a_(15.6)	
Without	153_a_(56.5)	332_b_(35.7)	14_a,b_(51.9)	138_c_(23.7)	
**Considered dropping out once**	26_a_(9.6)	100_a_(10.8)	7_a,b_(25.9)	186_b_(32.2)	< 0.01

B (+) Burnout positive; B (−) Burnout negative; D (+) Depressive symptoms positive; D (−) Depressive symptoms negative

Subscript letters a, b, c: If the mark letters were the same between any two groups, the difference between the two groups was not statistically significant. If the two groups of markers had different letters, the difference between the two groups was statistically significant

### Multinomial analysis

Multinomial logistic regression results comparing students without burnout or depressive symptoms with those who suffered only from burnout showed that being married (OR = 0.44, 95% CI 0.29–0.69) was the protective factor for those in the latter group. However, having children (OR = 1.79, 95% CI 1.17–1.74) and career choice regret (OR = 2.62, 95% CI 1.56–3.28) had a higher risk for students with only burnout. Moreover, students with only burnout who reported career choice regret neutral (OR = 2.98, 95% CI 1.84–4.80) were also at higher risk. Similar factors and results also appeared in the comparison between unaffected students and students with overlapping symptoms of burnout and depression. Furthermore, low family income (OR = 2.33, 95% CI 1.05–5.18) and thoughts of dropping out of school (OR = 2.62, 95% CI 1.55–4.43) imposed a higher risk tendency only among students with overlapping symptoms of burnout and depression. The academic year factor was different in the other categories compared with unaffected students; almost all other school years were deemed more serious burnout and/or depression symptoms than the first year master’s degree, but not all grades showed significant differences (see Table 2).

**Table 2:** Multinomial analysis of medical students’ burnout and (or) depressive symptoms

B (−) and D (−) [Reference]	Variables	OR (95% CI)	***P*** value
**B (+) and D (−)**^**a**^	**Marital status, Married**	0.44 (0.29–0.69)	**< 0.01**
	**Have children**	1.79 (1.17–1.74)	**< 0.01**
	**Career choice regret**		
	With	2.26 (1.56–3.28)	**< 0.01**
	Neutral	2.98 (1.84–4.80)	**< 0.01**
	Without	1 (Reference)	
	**Academic year**		
	Second-year, master’s degree	1.23 (0.80–1.90)	0.34
	Third-year, master’s degree	0.98 (0.65–1.46)	0.90
	First-year, doctor’s degree	3.89 (1.10–13.75)	**0.03**
	Second-year, doctor’s degree	1.47 (0.54–4.01)	0.45
	Third-year, doctor’s degree	2.39 (0.76–7.48)	0.14
	First-year, master’s degree	1 (Reference)	
**B (−) and D (+)**^**b**^	**Academic year**		
	Second-year, master’s degree	9.01 (1.03–78.62)	0.47
	Third-year, master’s degree	5.44 (0.64–46.32)	0.12
	First-year, doctor’s degree	56.23 (3.39–933.60)	**< 0.01**
	Second-year, doctor’s degree	12.47 (0.57–271.17)	0.11
	Third-year, doctor’s degree	–	–
	First-year, master’s degree	1 (Reference)	
**B (+) and D (+)**^**c**^	**Family income (yuan per month)**		
	< 5000	2.33 (1.05–5.18)	**0.04**
	5000–10,000	2.15 (0.96–4.85)	0.06
	10,000–15,000	1.63 (0.62–4.27)	0.32
	> 15,000	1 (Reference)	
	**Scores of postgraduate entrance examination**		
	< 300	0.87 (0.45–1.69)	0.67
	300–330	1.87 (1.05–3.34)	**0.03**
	330–360	1.39 (0.76–2.56)	0.28
	> 360	1 (Reference)	
	**Marital status, Married**	0.55 (0.34–0.89)	**0.02**
	**Have children**	1.97 (1.24–3.14)	**< 0.01**
	**Career choice regret**		
	With	3.87 (2.56–5.86)	**< 0.01**
	Neutral	3.56 (2.06–6.16)	**< 0.01**
	Without	1 (Reference)	
	**Considered dropping out once**	2.62 (1.55–4.43)	**< 0.01**
	**Academic year**		
	Second-year, master’s degree	2.33 (1.43–3.81)	**< 0.01**
	Third-year, master’s degree	1.32 (0.82–2.12)	0.25
	First-year, doctor’s degree	3.21 (0.79–13.00)	0.10
	Second-year, doctor’s degree	2.50 (0.84–7.43)	0.10
	Third-year, doctor’s degree	3.69 (1.08–12.61)	**0.04**
	First-year, master’s degree	1 (Reference)	

Dependent variables: symptoms of burnout and (or) depression

*Abbreviations*: *CI* Confidence interval, *OR* Odds ratio, *B(+)* Burnout positive, *B (−)* Burnout negative, *D (+)* Depressive symptoms positive, *D (−)* Depressive symptoms negative

^a^adjusted by degree type, family income, postgraduate entrance examination scores, hours worked or studied per week, hours slept per day, whether worked part-time once, view on current medical environment, considered dropping out once

^b^adjusted by degree type, family income, postgraduate entrance examination scores, hours worked or studied per week, hours slept per day, marital status, whether have children, whether worked part-time once, view on current medical environment, career choice regret, considered dropping out once

^c^adjusted by degree type, hours worked or studied per week, hours slept per day, whether worked part-time once, view on current medical environment

## Discussion

This nationwide study was one of the first to study the overlap and differences between burnout and depressive symptoms among Chinese medical students. We found the proportion of overlapping burnout and depression symptoms among the neurology graduate students accounting for nearly a third of the total number of participants, and for the characteristics of influencing factors in the overlap cases.

Studies on burnout and depressive have shown that their relationship is controversial and have provided convincing arguments for all sides of the issue [32–35]. Some scholars believe that burnout and depression are not isomorphic [32]. One view is that, although burnout and depression are closely related, they can be distinguished from each other [33]. Additionally, burnout is limited to work-related situations, while depression is more general [33]. On the other hand, some researchers suggest that burnout is a state of depression rather than an independent entity [34]. Furthermore, some studies suggest that evidence for the validity of the difference between burnout and depression is weak, and that burnout may reflect a “classic” depression process [35]. In brief, these studies point to possible overlap and differences between burnout and depression. In this study, burnout and depressive symptoms were not identical among medical students. The overlap of burnout and depressive symptoms was significant, but the rate was moderate in medical students.

Our findings indicate that almost all the students with depressive symptoms also had burnout, and that the ratio of symptoms in each of the four groups differed widely. Consistent with previous studies, the whole proportion of burnout was higher than that of depressive symptoms [12, 13]. Although previous studies have shown high levels of burnout and depressive symptoms among medical students [36], as well as a strong interplay between burnout and depression [37], they fail to mention that the majority of students with depressive symptoms also suffer from burnout. In fact, in one study, 83.1% of patients with depression experienced a high level of burnout, and whether they had experienced burnout differed significantly among those individuals [5]. While similar conclusions have been confirmed in studies of overlapping burnout and depressive symptoms in other occupations [38], and other studies have mentioned these overlapping symptoms among medical students [39], the symptom proportions in this relationship were confirmed in the present study. Our results illustrated not only the degree of overlap between burnout and depressive symptoms, but also the tendency to categorize students with depressive symptoms as also burnout students. According to one study, burnout symptoms are more common than depressive symptoms, and the overlap phenomenon is only moderate [40]. On the whole, our study seems to demonstrate this situation, and to hint that depressive symptoms in neurology graduate students may be more related to burnout, which indicates that it is important to assess graduate students for burnout.

In our study, students without burnout or depressive symptoms were used as a reference. The results of multinomial logistic regression showed that the students with only burnout and students with overlapping burnout and depressive symptoms had the same four associated factors. The four factors are similar to those reported in research results for medical interns, but are not exactly the same [41]. Among the comparable factors, being married is protective, consistent with the results of this study; however, having children is a risk factor, in contrast to our findings [41]. Possible reasons for this are the inconsistencies between factors across the two studies and the differences in their populations. Another important reason may be strong social pressure among Chinese young people, which has meant increasingly more of them choosing to marry later and having fewer children [42]. Many families, moreover, cannot afford to have children, and, clearly, graduate students who have no source of income cannot afford to this either. One study found a negative correlation between being married and burnout, possibly because of differences in study subjects [43]. In this study, being married was a protective factor. It may be that marriage plays a greater role in providing emotional support to young couples.

In this study, we observed more related factors that influenced the overlap of students’ burnout and depressive symptoms. Some studies assert that burnout is a state of depression, given the genesis of the burnout construct and the overlap of burnout and depression [44]. While this overlap was obvious among neurology graduate students in the present study, there was a significant overlap in the predictors for only burnout and for burnout–depressive symptoms overlapped. Importantly, the proportions of symptoms in each of the two groups was high, and there was a large differentiation. That is, many students who are burned out do not evidence the diagnostic standard for depressive symptoms. Among students with only depressive symptoms, there was only one significant influencing factor which was probably caused by the sample size of this group being too small to obtain more accurate results. The significant influence of academic year on students’ burnout and/or depressive symptoms was reflected in different academic year, which may have been caused by its instability. One survey of burnout among medical undergraduates showed that sophomores and juniors tend to be in the higher level than freshmen [45, 46], while another survey on medical graduate students’ burnout demonstrates significant differences among different years, with the third year being in the highest level [45, 47]. Most medical postgraduates in China are required to publish articles, and the increased pressure to complete their studies is likely to impose more stress for senior students.

Burnout and depressive symptoms have long been associated with medical students’ lived experience. College students in China live in an exam-oriented education system, which makes learning itself a huge source of pressure [48]. Therefore, relief from burnout and depressive symptoms should be integrated into the daily management of medical graduate students. This study illustrated that a high proportion of the neurology graduate student survey sample evidenced an overlap of burnout and depressive symptoms. Moreover, these students with lower mental health level were more likely to be affected by daily life and/or study. Thus, it is urgent to focus on this group. Further study on overlapping symptoms may provide a new focus for educational administration in their management of students. Studies have shown that higher levels of burnout are associated with reduced help-seeking behavior among people with mental health problems, who may not only suffer from burnout and depressive symptoms, but may also be reluctant to seek treatment owing to psychological pressures such as a sense of shame and fear of disclosure [8, 49]. Additionally, many people may underestimate the severity of the symptoms and may not seek help when they think they are “burned out” [50]. The present study found a tendency for depressive symptoms to be included in the burnout subgroup, and also found that the influencing factors were similar. It is suggested that improvement measures in burnout or depressive symptoms can affect all of these students, thus alleviating their burnout and depressive symptoms may eventually become more efficient. For this reason, it is important to focus on the overlap between burnout and depressive symptoms.

Education departments should pay more attention to adjustments in medical students’ curriculum and leisure activities, so students can combine work with rest. University administrators should improve the prevention of students’ negative psychological issues, help students manage them, and pay attention to vulnerable groups. Furthermore, increased action should be taken and more time spent communicating with students who evidence overlapping burnout and depressive symptoms instead of waiting for them to ask for help. It is recommended that students’ depressive symptoms and burnout be assessed concurrently, and that students with serious results be given appropriate medications and/or psychological intervention. Finally, it is essential that sensitive words such as “stigma” are avoided, to reduce the potential for causing students harm.

### Limitations

First, this study was a cross-sectional design and was limited to graduate students in neurology, thus restricting the external validity of the study that we were unable to assess any causal relationship. The results should be validated using longitudinal methods in future studies. Second, only self-reported questionnaires were used for evaluation. Third, this study was not administered using a random sampling methods may cause an indeterminate response rate. Fourth, the information collected by the self-report questionnaire had certain limitations, such as marital tension, physical activity, social support and personality traits, which were not reflected in this study. Finally, although prime-MD is widely used to assess depressive symptoms, it may not be sufficient to measure the intensity of depressive symptoms. In subsequent studies, more comprehensive measurement methods may be needed to further support the results of this study.

## Conclusions

This study identified an obvious overlap ratio of burnout and depressive symptoms among Chinese neurology graduate students. Some similarities and differences in related influencing factors were found, which indicates that the occurrence of depressive symptoms among medical students is closely related to whether they are burned out. Further, burnout may be a pre-depression state. While burnout often occurs alone, depressive symptoms do not typically occur alone, which provides more insights into and evidence about the mental health of medical students.

## Supplementary Information

**Additional file 1.**

## Data Availability

The datasets generated during and analyzed during the current study are not publicly available due to the data confidentiality requirements of Chinese Medical Doctor Association but are available from the corresponding author on reasonable request.
