# Supplementary material for: Overlap of burnout-depression symptoms among Chinese neurology graduate students in a national cross-sectional study
Source: BMC Med Educ. 2021 Feb 2;21:83. doi: 10.1186/s12909-021-02511-3 (PMC7851928; doi:10.1186/s12909-021-02511-3)
Supplement: Supplementary file 1 — Additional file 1. [file 12909_2021_2511_MOESM1_ESM.docx]

The current situation of Chinese neurology graduate students:

the survey of China Neurologist Association

Dear sir/madam:

The aim of this study is to promote the gradual deepening of medical graduate education, and also hope to provide more targeted services. This survey is conducted on a voluntary and anonymous basis. If you complete this questionnaire, you will be deemed to have given informed consent. Please fill in your true information and opinions. Thank you very much for your participation.

Thank you for taking part in this questionnaire, and wish you a successful study!

China Neurologist Association

**1. Basic personal information:** **(please put the "√" in brackets where appropriate, or fill in the __)**

1.1 Gender :( ) male ( ) female

1.2 Your academic year :

( ) master first year ( ) master second year ( ) master third year ( ) doctor first year ( ) doctor second year ( ) doctor third year

1.3 Your age: ____________

1.4 Degree type: ( ) Clinical practice ( ) Academic practice

1.5 Total household income per month (yuan):

( ) < 5,000 ( ) 5,000–10,000 ( ) 10,000–15,000 ( ) > 15,000

1.6 Scores of postgraduate entrance examination:

( ) < 300 ( ) 300–330 ( ) 330–360 ( ) >360

1.7 Hours worked or studied per week (hours):

( ) < 35 ( ) 35–45 ( ) 45–55 ( ) >55

1.8 Hours slept per day (hours):

( ) < 6 ( ) 6-8 ( ) 8-10 ( ) >10

1.9 Current marital status:

( ) Married ( ) single

1.10 Do you have children:

( ) Yes ( ) No

1.11 Have you taken part time jobs since your master's or doctor's degree:

( ) Yes ( ) No

**2. Please fill in the following questions truthfully. The following questions are all single choice.**

2.1. In general, how do you like your health in the past month? ( )

A. Very good B. Good C. Ok D. Not bad E. Poor F. Very bad

2.2 In the past month, have you restricted your normal physical activities (such as walking, shopping, or climbing stairs) due to a physical health problem? ( )

A. No B. Very few C. Sometimes D. Quite a lot E. Can't activities

2.3 In the past month, whether at home or outdoors, have you been unable to perform daily tasks (such as cleaning, washing, cooking) due to physical health problems? ( )

A. No B. A little C. Some D. Quite A lot E. Can't do

2.4 Have you felt much physical pain in the past month? ( )

A. No B. very mild C. mild D. moderate E. severe F. Very serious

2.5 How many times in the past month have you felt energized? ( )

A. Very often B. Often C. Not too often D. Occasionally E. No

2.6 In the past month, how many times have you reduced your social activities (such as gatherings with family or friends) due to physical health or emotional problems? ( )

A. Not B. A little C. Some D. Quite a lot E. Can't socialize

2.7 How many times in the past month have you experienced emotional problems (such as anxiety, depression, or irritability)? ( )

A. not at all B. rarely C. Sometimes D. often E. Very often

2.8 How many times in the past month have emotional issues prevented you from going to work, school, or other daily activities? ( )

A. not at all B. rarely C. Sometimes D. often E. Very often

2.9 Do you feel exhausted from your study and work? ( )

A. Never B. a few times a year or less C. once a month or less D. several times a month E. once a week F. a few times a week G. every day

2.10 You feel exhausted after a day of study and work: ( )

A. Never B. a few times a year or less C. once a month or less D. several times a month E. once a week F. a few times a week G. every day

2.11 Do you feel tired when you have to get up in the morning to face a day of study or work? ( )

A. Never B. a few times a year or less C. once a month or less D. several times a month E. once a week F. a few times a week G. every day

2.12 Do you think you can understand the patient's feelings more easily? ( )

A. Never B. a few times a year or less C. once a month or less D. several times a month E. once a week F. a few times a week G. every day

2.13 Do you think you can treat patients objectively? ( )

A. Never B. a few times a year or less C. once a month or less D. several times a month E. once a week F. a few times a week G. every day

2.14 Is being around people all day a real stress for you? ( )

A. Never B. a few times a year or less C. once a month or less D. several times a month E. once a week F. a few times a week G. every day

2.15 Can you effectively deal with the patient's problems? ( )

A. Never B. a few times a year or less C. once a month or less D. several times a month E. once a week F. a few times a week G. every day

2.16 Are you tired of study or work? ( )

A. Never B. a few times a year or less C. once a month or less D. several times a month E. once a week F. a few times a week G. every day

2.17 Do you think you can bring positive energy to others through your study or work? ( )

A. Never B. a few times a year or less C. once a month or less D. several times a month E. once a week F. a few times a week G. every day

2.18 Have you become more impersonal since you have been in this industry? ( )

A. Never B. a few times a year or less C. once a month or less D. several times a month E. once a week F. a few times a week G. every day

2.19 Are you worried that this industry or job is making you colder and colder? ( )

A. Never B. a few times a year or less C. once a month or less D. several times a month E. once a week F. a few times a week G. every day

2.20 Do you feel energetic? ( )

A. Never B. a few times a year or less C. once a month or less D. several times a month E. once a week F. a few times a week G. every day

2.21 Are you frustrated by your study or work? ( )

A. Never B. a few times a year or less C. once a month or less D. several times a month E. once a week F. a few times a week G. every day

2.22 Do you think you study or work too hard? ( )

A. Never B. a few times a year or less C. once a month or less D. several times a month E. once a week F. a few times a week G. every day

2.23 Don't you really care what happens to the patient? ( )

A. Never B. a few times a year or less C. once a month or less D. several times a month E. once a week F. a few times a week G. every day

2.24 Does working directly with patients give you a lot of stress? ( )

A. Never B. a few times a year or less C. once a month or less D. several times a month E. once a week F. a few times a week G. every day

2.25 When you are with a patient, is it easy for you to create a relaxed environment? ( )

A. Never B. a few times a year or less C. once a month or less D. several times a month E. once a week F. a few times a week G. every day

2.26 Do you feel a sense of accomplishment after working closely with your patients? ( )

A. Never B. a few times a year or less C. once a month or less D. several times a month E. once a week F. a few times a week G. every day

2.27 Have you done a lot of meaningful things in this job? ( )

A. Never B. a few times a year or less C. once a month or less D. several times a month E. once a week F. a few times a week G. every day

2.28 Do you feel physically and mentally exhausted while studying or working? ( )

A. Never B. a few times a year or less C. once a month or less D. several times a month E. once a week F. a few times a week G. every day

2.29 Can you deal with emotional problems calmly in your study or work? ( )

A. Never B. a few times a year or less C. once a month or less D. several times a month E. once a week F. a few times a week G. every day

2.30 Do you think patients will blame you for some of their problems? ( )

A. Never B. a few times a year or less C. once a month or less D. several times a month E. once a week F. a few times a week G. every day

2.31 In the last month, did you often feel in low spirits, depressed or hopeless? ( )

A. Yes B. No

2.32 In the last month, were you often troubled by the feeling that you were not interested or had no fun in doing things? ( )

A. Yes B. No

2.33 Have you ever thought about taking your own life? ( )

A. Yes B. No

2.34 Have you ever thought about taking your own life in the past year? ( )

A. Yes B. No

2.35 Have you ever tried to end your own life? ( )

A. Yes B. No

2.36 Are you planning to become a doctor after graduation? ( )

A. Won't be a doctor B. Doesn't matter C. Will be a doctor

2.37 Are you planning to choose another department after graduation? ( )

A. Won't choose other departments B. Doesn't matter C. will choose other departments

2.38 What do you think of the current medical environment? ( )

A. Good B. Neutral C. Poor

2.39 If you can choose again, will you still choose to study medicine? ( )

A. Yes B. Neutral C. No

2.40 Have you ever thought about dropping out? ( )

A. Yes B. No
